# Desialylation is associated with apoptosis and phagocytosis of platelets in patients with prolonged isolated thrombocytopenia after allo-HSCT

**DOI:** 10.1186/s13045-015-0216-3

**Published:** 2015-10-23

**Authors:** Xiao-Hui Zhang, Qian-Ming Wang, Jia-Min Zhang, Fei-Er Feng, Feng-Rong Wang, Huan Chen, Yuan-Yuan Zhang, Yu-Hong Chen, Wei Han, Lan-Ping Xu, Kai-Yan Liu, Xiao-Jun Huang

**Affiliations:** Peking University People’s Hospital, Peking University Institute of Hematology, No. 11 Xizhimen South Street, Xicheng District, Beijing, 100044 People’s Republic of China

## Abstract

**Background:**

Prolonged isolated thrombocytopenia (PT) is a frequent complication in patients who undergo allogeneic hematopoietic stem cell transplantation (allo-HSCT), and it is associated with an adverse prognosis. In this study, we hypothesized that desialylation on platelet surfaces was associated with PT after allo-HSCT. The mechanisms participating in this process may include NEU1 translocation, platelet apoptosis, and phagocytosis by macrophages.

**Methods:**

PT was defined as a peripheral platelet count less than 100 × 10^9^/L without sustained anemia or leukopenia for more than 3 months after allo-HSCT. 34 patients were identified consecutively from a cohort of 255 patients who underwent allo-HSCT for hematologic malignancies between May and October 2014 at Peking University Institute of Hematology. Desialylation, enzyme expression, and phagocytosis were detected using flow cytometry, immunofluorescence, RT-PCR, Western blot, and so on.

**Results:**

Platelets from the PT patients had significantly fewer sialic acids (*P* = .001) and increased β-galactose exposure indicative of desialylation on the surface (*P* = .042), and serum from the PT patients showed a higher sialic acid concentration (8.400 ± 0.2209 μmol/L, *P* < .001). The sialidase NEU1 was over-expressed from mRNA to protein levels, and its catalytic activity was increased in platelets from the PT patients. Desialylation of GPIbα in the PT patients was correlated with changes in 14-3-3ζ distribution, which, relative to Bad activation, modulated the expression of Bcl-2 family proteins, depolarized the inner membrane of the mitochondria, and initiated the intrinsic mitochondria-dependent pathway of apoptosis. Macrophages derived from the THP-1 cell line preferred to phagocytize desialylated platelets from the PT patients in vitro*.* We also revealed that oseltamivir (400 μmol/L) could inhibit 50 % of the sialidase activity on platelets and could protect 20 % of platelets from phagocytosis in vitro*.*

**Conclusions:**

Desialylation of platelets was associated with platelet apoptosis and phagocytosis, whereas oseltamivir could reduce platelet destruction in the periphery, indicating a potential novel treatment for PT after allo-HSCT.

**Electronic supplementary material:**

The online version of this article (doi:10.1186/s13045-015-0216-3) contains supplementary material, which is available to authorized users.

## Introduction

Prolonged isolated thrombocytopenia (PT), which is defined as a consistently low platelet count for more than 3 months after transplantation despite the recovery of all other peripheral blood cell lines, is a frequent complication of allogeneic hematopoietic stem cell transplantation (allo-HSCT), with an incidence of 5–37 % [1–4]. Previous studies have suggested that poor platelet recovery following allo-HSCT is related to poor overall survival and has a significant association with III–IV degree GvHD, resulting in an adverse patient prognosis [3–8].

Although the exact pathogenesis of PT remains unknown, there are two primary theories as to its origin: destruction in the peripheral circulation and deficiency of production in the bone marrow [1, 2, 9, 10]. In the bone marrow, our previous study found that cellular elements in the bone marrow microenvironment, including endothelial cells and perivascular cells, were significantly reduced in PT patients, indicating that an impaired bone marrow vascular microenvironment might contribute to the occurrence of PT [9]. Another of our studies showed that increased CD8+/CX3CR1+ T cells in the bone marrow might also be associated with PT [11]. However, little research has explored the increased turnover of platelets in the peripheral circulation. It has been demonstrated that some morphological platelet indices, such as the mean platelet volume (MPV), have value for distinguishing hyperdestructive from hypoproductive thrombocytopenia [12, 13]. Enlarged MPV can be used as a measurement of young platelets, indicating that the origin of thrombocytopenia was peripheral destruction [13]. Other laboratory methods, such as plasma glycocalicin index (GCI), have been reported to be useful for the diagnosis of thrombocytopenia. Glycocalicin is a carbohydrate-rich hydrophilic fragment, and the GCI (plasma glycocalicin level normalized to the individual platelet count) reflects the rate of platelet destruction [12]. Previous studies have demonstrated an association between a high GCI and PT after HSCT [2], indicating increased platelet clearance and turnover in PT pathogenesis*.*

Our known platelet clearance mechanisms include antibody-mediated clearance by spleen macrophages, as observed in immune thrombocytopenia (ITP), and platelet consumption due to massive blood loss [14]. However, the anti-GPIIb/IIIa antibody response failed to show a necessary association with PT after HSCT via platelet destruction in the periphery, and some recipients with PT have neither anti-GPIIb/IIIa antibody detected nor massive platelet consumption [2]. Therefore, there must be some antibody-independent platelet clearance pathogenesis in PT patients. It has been revealed that Fc-independent platelet hepatic clearance was observed in some ITP patients, especially those with anti-GPIb/IX antibodies [15–17]. These patients showed poor responses to steroids and IVIG treatment, indicating an Fc-independent platelet clearance mechanism [18, 19]. Li et al. reported that platelet desialylation (depletion of sialic acids) occurred in the presence of anti-GPIbα antibodies, and desialylation of GPIbα occurred directly upstream of platelet activation and apoptosis [20]. Moreover, desialylation of the platelet surface membrane, especially GPIbα, has been demonstrated to be a key step in the clearance of chilled platelets after transfusion [21–24].

Sialic acids are monosaccharides with a shared 9-carbon backbone, and they are typically found at the outermost end of the glycan chains in all cell types. Sialic acids play pivotal roles in many physiologically and pathologically important processes. They play numerous roles in several aspects of immunity, and they might serve as “self” markers in different physiological and pathophysiological functions [25–27]. Desialylation of GPIbα, which is mediated by human sialidase NEU1, can induce exposure of β-galactose and β-*N*-acetyl-D-glucosamine (β-GlcNAc), which can be recognized by either macrophages or liver cells and can trigger phagocytosis of chilled platelets [22, 24, 28, 29].

A prospective cohort study was conducted to determine whether desialylation of the platelet surface played a role in the pathogenesis of PT after allo-HSCT. We measured the sialic acid content and desialylation markers on platelet surface membranes from PT patients and HSCT recipients with normal blood cell counts. We also tested sialidase expression and activity from platelets and analyzed apoptotic pathways, as well as phagocytosis by macrophages. Here, we demonstrate that the platelet surfaces were desialylated by NEU1 in PT patients, which was associated with platelet apoptosis and phagocytosis by macrophages in the pathogenesis of PT. Moreover, the sialidase inhibitor oseltamivir could inhibit NEU1 activity and reduce phagocytosis of platelets from PT patients in vitro.

## Materials and methods

### Patients and controls

PT was defined as a peripheral platelet count less than 100 × 10^9^/L without sustained anemia or leukopenia for more than 3 months after allo-HSCT. Patients were identified consecutively from a cohort of 255 patients who underwent allo-HSCT for hematologic malignancies between May and October 2014 at Peking University Institute of Hematology. Patients with clear causes of thrombocytopenia were excluded. These causes included engraftment failure, recurrence of the underlying malignancy, systemic infection, II–IV degree aGvHD, cytomegalovirus (CMV) reactivation, and drugs after allo-HSCT. A total of 34 patients who had developed PT after allo-HSCT were eligible for our study; 26 allo-HSCT recipients who had recovered all types of cell counts after day 90 were extracted as controls from the same cohort. Blood samples from ten healthy individuals were also used as healthy control subjects. All of the samples were collected after the patients provided written informed consent according to the local ethics policy guidelines and the Declaration of Helsinki.

### Transplantation protocols

Pretransplantation conditioning was performed with cytarabine (4 g/m^2^ per day, days −10 to −9), busulfan (3.2 mg/kg per day, intravenously days −8 to −6), cyclophosphamide (1.8 g/m^2^ per day, days −5 to −4), and semustine (250 mg/m^2^, day −3) and either with or without rabbit ATG (thymoglobulin; Imtix Sangstat, Lyon, France, 1.5 mg/kg per day, days −5 to −2). GvHD prophylaxis was implemented with cyclosporine A (CsA), mycophenolate mofetil (MMF), and short-course methotrexate (MTX) as previously described [30–33]. The effective concentration range of CsA is 150–300 ng/mL. Oral MMF was administered in doses of 0.5–1.0 g/day from day −1 to day 30 after transplantation. MTX was administered i.v. at doses of 15 mg/m^2^ on day 1 and 10 mg/m^2^ on days 3, 6, and 11 [30, 31]. Grafts were granulocyte-colony stimulating factor-mobilized bone marrow and blood cells as previously described [31, 34]. Oral acyclovir, sulfamethoxazole, and norfloxacin were administered for infection prophylaxis [32, 35]. Ganciclovir was used from day −9 to day −2 before transplantation for the prophylaxis of CMV infection [36, 37]. Antifungal agents were administered as previously described [38]. Bone marrow aspiration and cytogenetic studies were performed after transplantation to assess engraftment, as described in our previous studies [1, 9, 31, 34, 39, 40].

### Clinical definition and evaluation

Engraftment was demonstrated by increasing neutrophil and platelet counts unsupported by transfusions. Risk stratification prior to transplantation was evaluated as previously described [9, 34]. GvHD was scored as either acute or chronic based on published criteria [41–43]. A history of GvHD was defined as patients who were diagnosed with either grade II–IV acute GvHD or extensive chronic GvHD and who required treatment [1, 2, 44]. History of CMV reactivation was defined as either positive twice for plasma CMV via PCR or a diagnosis of CMV disease [1].

### Platelet sample preparation

Venous blood was obtained from the subjects by venipuncture and was drawn into 0.1 M citrate-based anticoagulant (BD Vacutainer). Platelet-rich plasma (PRP) was prepared by centrifugation at 125×*g* for 20 min, and the platelets were separated from PRP by centrifugation for 5 min at 850×*g* in a buffer containing 1 μg/mL prostaglandin E1 [28]. Fresh platelets were resuspended at 10^7^/mL.

### Measurement of sialic acid residues and glycan exposure on platelet surface

Platelet surface sialic acid residues and glycan exposure were evaluated by the binding of fluorescein-labeled lectins by flow cytometry [22, 24, 29, 45, 46]. The serum sialic acid concentration and sialidase activity were measured using ELISA kit (Jrdun Biotech). Desialylation of GPIbα was confirmed by immunoblot analysis. Sialidase expression and location were tested by RT-PCR, flow cytometry, and immunofluorescence. Sialidase activity assays were performed as previously described [28].

### Immunofluorescence and microscopy

The platelets were either fixed in BD Cytofix or fixed and permeabilized in BD Cytofix/Cytoperm (22 °C, 20 min), followed by centrifugation onto poly-L-lysine-coated coverslips (500×*g* for 2.5 min). Cells were blocked as previously described [47]; the platelets were incubated overnight with rabbit anti-NEU1 IgG Ab (Santa Cruz Biotechnology) and then were washed in triplicate with phosphate-buffered saline (PBS). The washed platelets were incubated for 1.5 h at room temperature with the secondary Ab conjugated with either Alexa Fluor 488 or 568 (Molecular Probes) at a dilution of 1:500, followed by three washes with PBS. Images were obtained as previously described [28].

### Apoptosis assay

Bax and Bcl-xL were measured by flow cytometric analysis. 14-3-3ζ association was detected by immunoprecipitation. For determination of the mitochondrial membrane potential (ΔΨm), 100 μL of platelet suspension was incubated with JC-1 (0.5 μM, 30 min, 37 °C). In viable cells, a high ΔΨm promotes the directional uptake of JC-1 into the matrix, where JC-1 forms J-aggregates (λex 490 nm, λem 570–610 nm). In apoptotic cells, a low ΔΨm preserves the monomeric form (λex 490 nm, λem 535 nm). Changes in the ΔΨm were expressed as the ratio of the platelets in the lower-right to the upper-right quartile [48].

### In vitro phagocytosis assay

THP-1 monocytic cell lines were cultured to a density of (1–2) × 10^6^/mL in RPMI 1640, glutamine (2 mmol/L), penicillin (100 U/mL), and streptomycin (100 μg/mL) at 37 °C. Maturation was induced by 500 nmol/L phorbol myristate acetate (PMA) (24 h at 37 °C). Of platelet (PLT) suspension, 100 μL was labeled with 1 μmol/L mepacrine in Hepes-Tyrode (pH 7.2, 5 min, 22 °C). Mepacrine-labeled PLTs (10^7^/well) that were previously subjected to different treatments were added to the phagocytes in Ca^2+^- and Mg^2+^-containing HBSS (GIBCO Invitrogen) and were incubated for 30 min at 37 °C. The binding of PLTs to macrophages was expressed as the percentage of CD42b/CD14-positive particles to the total number of CD42b- and/or CD14-positive particles. Phagocytosis of PLTs by macrophages was measured by FACS analysis of mepacrine-positive CD14 cells that were inaccessible to the CD42b antibody, and it is expressed as the percentage of the total number of CD14-positive/CD42b-negative particles [49–51].

### Statistical analysis

All of the data are presented as the means ± SEMs unless otherwise indicated. All numeric data were analyzed for statistical significance by non-paired Student’s *t* test (unless otherwise specified) for multiple comparisons, using Prism software (GraphPad). *P* < .05 was considered statistically significant.

## Results

### Patient characteristics

We identified 34 consecutive patients with PT, whereas the comparison cohort consisted of 26 subjects after allo-HSCT. The patient characteristics are summarized in Table 1. All of the characteristics, except a history of CMV reactivation and a history of GvHD, were nearly equally represented.

**Table 1:** Clinical characteristics of the patients after HSCT with PT

Characteristics	PT cases (*n* = 34)	Controls (*n* = 26)	*P* value^a^
Time of evaluation (post-HSCT)	100 (80–150)	121 (87–149)	.078
Age at HSCT, median (range), year	27 (11–56)	26 (3–58)	.959
Gender (male/female)	22/12	15/11	.603
PLT count (×10^9^/L)	33.68 (12–83)	143.38 (100–219)	<.001
Disease type			
Acute Leukemia	20	14	.095
Other	21	5	
Source of stem cell			
BM and PB	29	25	.221
PB	5	1	
Transplanted total nucleated cell dose, ×10^8^/kg	7.195 (5.00–10.10)	6.905 (3.44–9.12)	.350
Transplanted CD34+ cells, ×10^6^/kg	1.925 (0.73–7.42)	2.465 (0.38–4.47)	.685
Donor type			
HLA partially matched related donor	26	19	.689
Unrelated donor	2	1	
Identical sibling	6	6	
Status at HSCT			
Standard risk	26	20	.999
High risk	8	6	
Conditioning regimen			
BU/CY + ATG	27	19	.239
BU/CY	4	7	
TBI	3	0	
History of severe GvHD	21	8	.021
History of CMV reactivation	26	12	.029

^a^The continuous variables were compared using the one-way ANOVA, and the differences in frequency between the two groups were compared using the Chi-square test

A larger proportion of the PT patients had a history of grades II to IV acute GvHD or extensive chronic GvHD after transplantation (*P* = .021). Additionally, more allo-HSCT recipients with PT had experienced CMV reactivation from the day when they accepted the donors’ stem cells (*P* = .029).

There were no significant differences between the patients with PT and the controls (*P* > .05) with regard to the demographic and clinical characteristics, including age, sex, underlying disease, disease status before transplantation, donor types, source of stem cells, transplanted total nucleated cell dose, CD34+ cell dose, conditioning regimen, history of GvHD, history of CMV reactivation, and anti-CMV therapy (Table 1).

### Patients with PT have higher MPV and increased GCI

**Fig. 1:**
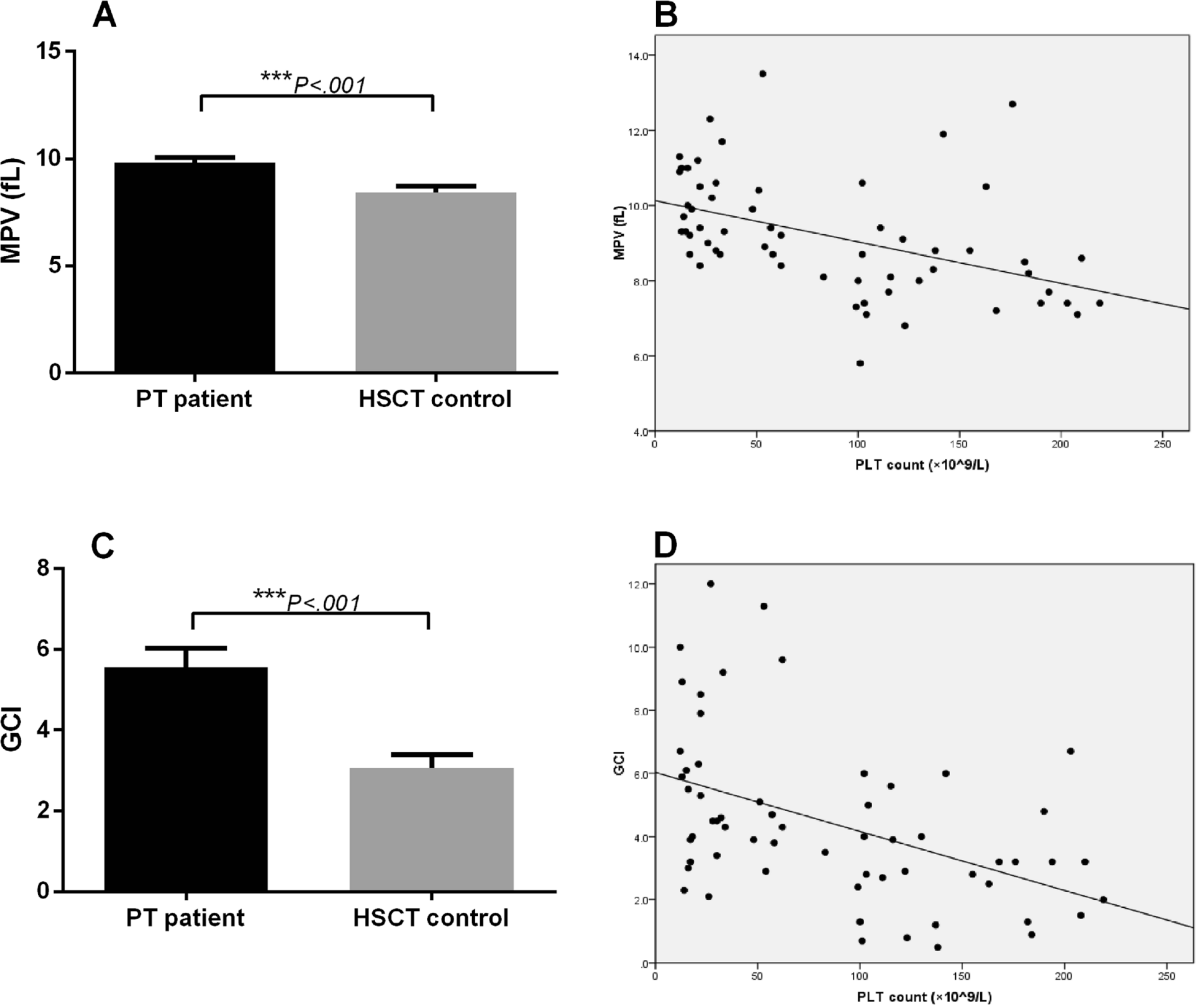
Platelets from PT patients showed hyperdestruction. **a** Histogram of MPV. MPV was significantly higher in PT patients. **b** The inverse correlation between MPV and PLT count. **c** Histogram of GCI. **d** The inverse correlation between GCI and PLT counts

To confirm that hyperdestruction in the peripheral blood was involved in the pathogenesis of PT, we measured the MPV and GCI of the PT patients and the control recipients. MPV was significantly higher in the PT patients (9.82 ± 1.31 vs. 8.41 ± 1. 54, *P* < .001), and an inverse correlation between platelet count and MPV value was demonstrated (*ρ* < 0.001, *r* = −0.56, Spearman’s rank correlation rho) (Fig. 1a, b). To evaluate further the platelet turnover, we calculated the GCI of the PT patients. GCI was markedly increased in the PT patients, compared to the control group (5.56 ± 2.68 vs. 3.06 ± 1.78, *P* < .001) (Fig. 1c). Taken together, the PT patients had significantly higher MPV and increased GCI, also indicating elevated platelet destruction.

### Platelets from patients with PT have higher desialylation levels

**Fig. 2:**
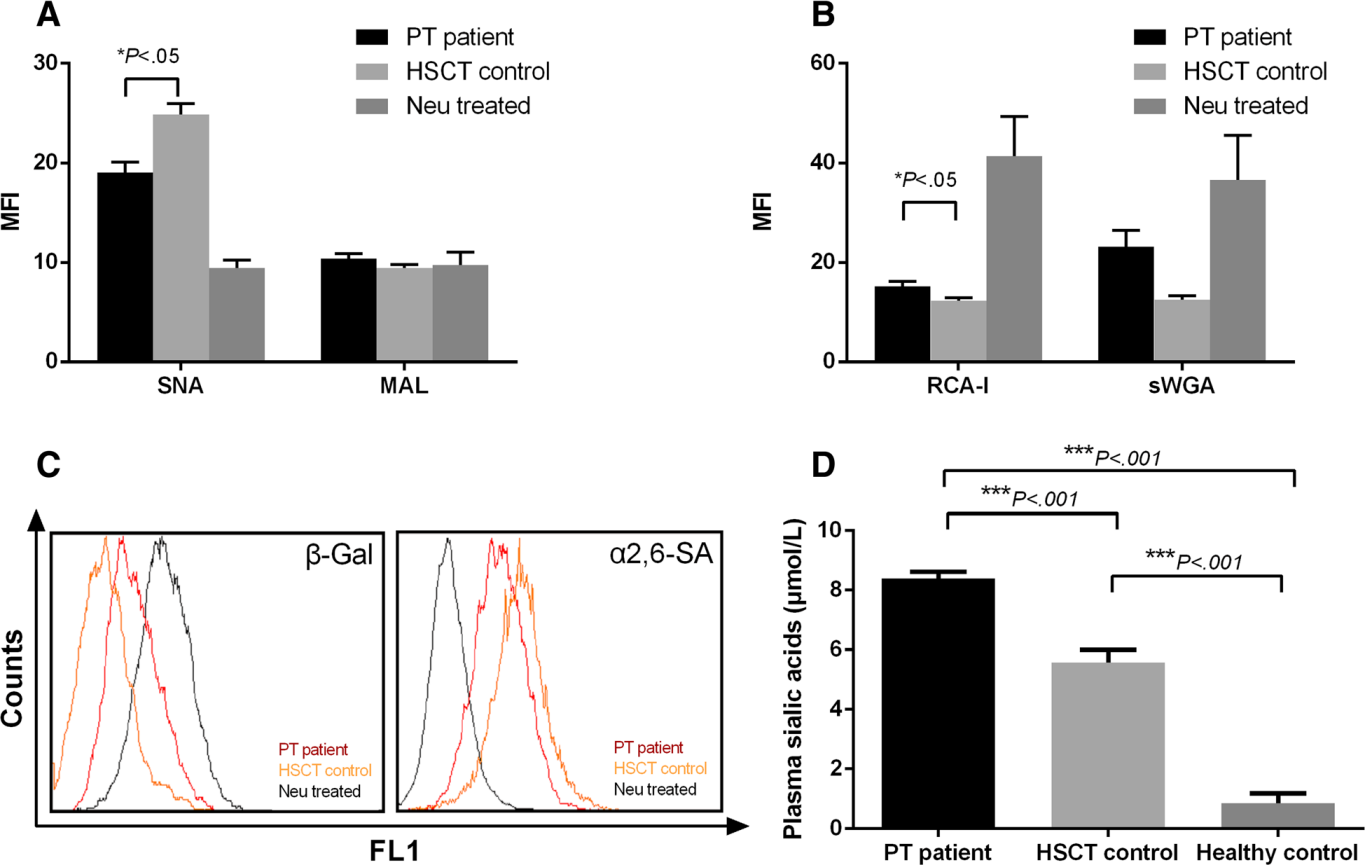
Sialic acid residues on platelet surfaces and in plasma. Lectin binding to fresh platelets was tested in the presence or in the absence of 10 mU of α2-3,6,8,9-sialidase from *A ureafacients* (Neu treated). **a** Flow cytometry analysis of sialic acid residues on platelet surfaces, as detected by FITC-labeled SNA or MAL. **b** β-galactose and β-GicNAc exposure on platelet surface glycoprotein, as detected with FITC-labeled RCA-I or succinyl-wheat germ agglutinin (sWGA), respectively. **c** Typical graph of flow cytometry analysis of platelet surface α2,6-sialic acid (α2,6-SA). **d** Serum sialic acid concentration measured by ELISA

Sialic acid moieties were detected by flow cytometry using a panel of lectins (Fig. 2a). In this study, the platelets from the PT patients showed significantly lower levels (*P* = .001) of binding to *Sambucus nigra* lectin (SNA), which is a lectin specific for the α2,6-sialic acid residues on the platelet membrane. However, the α2,3-sialic acid residues, measured by *Maackia Amurensis* lectin (MAL), showed no difference between the patient and control groups (*P* = .227).

With regard to desialylation markers, we observed that the β-galactose exposure levels were higher on the PT patient platelet surfaces, as detected by RCA-I (*P* = .042, Fig. 2b). In contrast, the levels of β-GlcNAc exposure showed no significant differences between the PT patient and control groups (*P* = .063).

The sialic acid concentration in the serum (Fig. 2c) from the patients with PT was higher than in the control group (8.400 ± 0.2209 μmol/L vs. 5.579 ± 0.4219 μmol/L, respectively, *P* < .001), but both concentration levels were higher than those of the healthy volunteers (0.8660 ± 0.3251 μmol/L, *N* = 10).

### GPIbα is the primary glycoprotein that is desialylated on the platelet membrane

We investigated the glycoprotein that was the primary substrate that was desialylated and that exposed β-galactose residues. Immunoblotting of desialylated platelet lysates from the PT patients using RCA-I binding showed multiple glycoproteins with exposed β-galactose residues. Two major desialylated proteins were shown, one of which had an apparent molecular weight of 140 kDa, a size consistent with GPIbα, which was confirmed by probing the lysates with a specific antibody. The other protein of 240 kDa was identified as a platelet-bound von Willebrand factor (vWF) (Additional file 1: Figure S2).

### Human sialidase NEU1 is over-expressed on the platelet surface

**Fig. 3:**
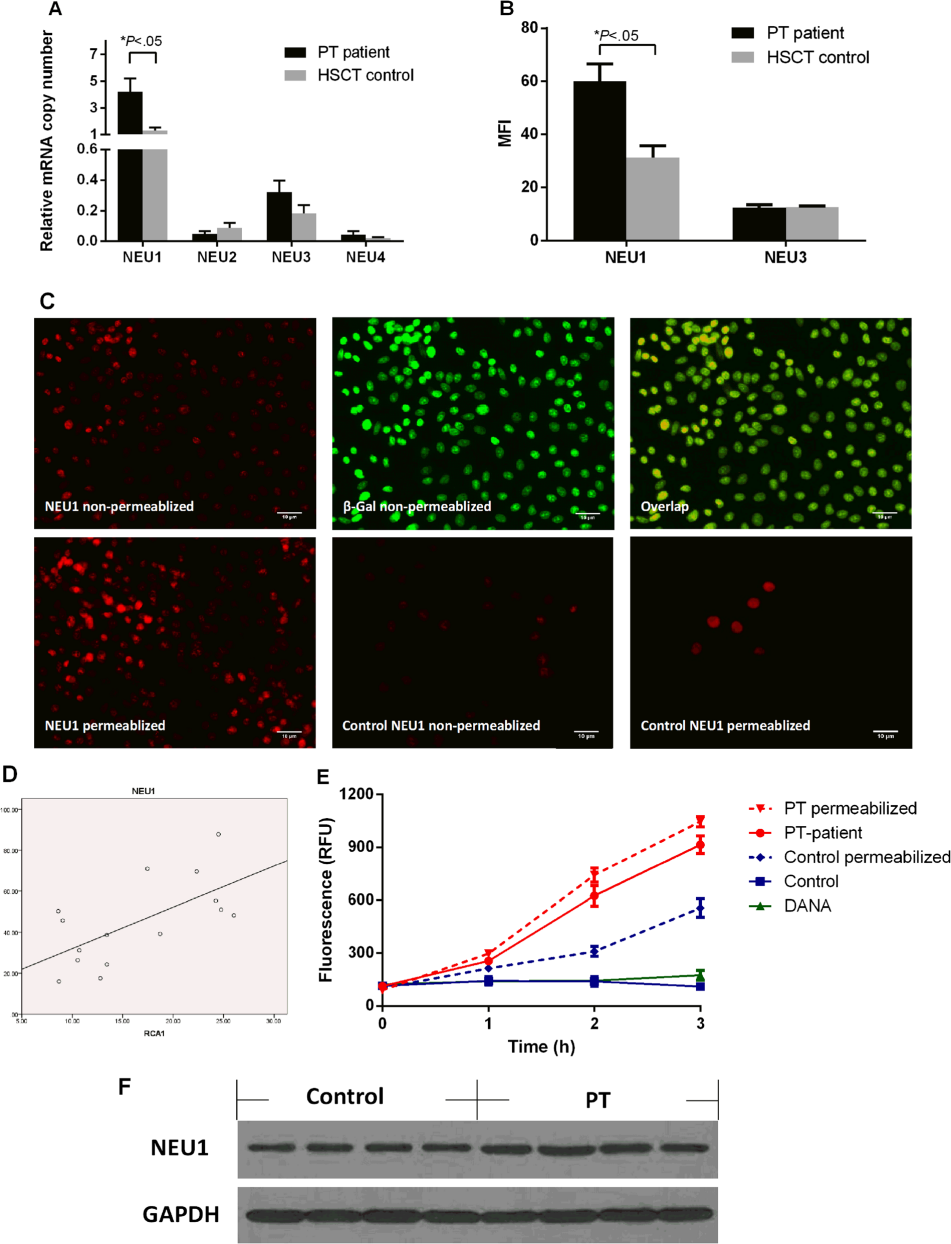
Sialidase expression and activity. **a** Total RNA isolated from platelets was reverse-transcribed, and the resulting cDNA was used as a template for amplification with primers corresponding to NEU1, NEU2, NEU3, and NEU4, as well as HPRT as a housekeeping gene control. The mRNA level for each sialidase was normalized to HPRT. **b** Flow cytometry analysis of NEU1 and NEU3 expression on platelet surfaces. The data are presented as MFI. **c** NEU1 localization in the platelets from the patients and the control group. The fluorescence intensity of NEU1 in the PT platelets was slightly elevated after permeabilization, and its distribution was correlated with that of β-Gal. The NEU1 signals of the platelets from the control group were weaker than those from the PT patients, especially when not permeabilized. **d** Correlation between the increase in RCA-I and NEU1 expression in the PT patients. *R* ^2^ = 0.408. Correlation between NEU1 and RVA-I MFI was determined by Spearman’s correlation coefficient; *P* = .585, *P* = .17. **e** Sialidase activity inside and on the surfaces of platelets from the PT patients. The total sialidase activity in platelets was measured after detergent permeabilization. **f** Total NEU1 expression of the platelets from the PT patients and the control group

To elucidate further the mechanism of desialylation, the expression of human sialidases, NEU1, NEU2, NEU3, and NEU4 was measured in the platelets from the PT patients. Sialidase expression was tested using flow cytometry, quantitative PCR (qPCR), and immunofluorescence microscopy. A real-time qPCR approach was adopted to detect mRNA for the four types of sialidases, and the data were normalized to HPRT mRNA in the same sample. NEU1 mRNA was significantly higher in platelets from the PT patients (4.212 ± 2.872 copies vs. 1.301 ± 0.678 copies, *P* = .049, Fig. 3a).

NEU1 expression was significantly higher (*P* = .002) on the membrane of the platelets from the control group as measured by flow cytometry and immunofluorescence, and its distribution was similar to that of the ECL binding experiments (Fig. 3b, c). We also observed a positive correlation between the increased levels of RCA-I binding and NEU1 surface expression (Spearman’s correlation *ρ* = 0.585, *P* = .017, Fig. 3d). The total NEU1 expression was measured after detergent permeabilization, which indicated that NEU1 translocated from the cytoplasm to the platelet surface (Fig. 3c).

A catalytic activity assay showed that platelets from the control group had little surface sialidase activity toward 4-MU-NeuAc, but this activity could be detected on the platelet surface from the PT patients. The total sialidase activity was higher in the platelets from the PT patients (Fig. 3e). All of these results suggested that NEU1 was responsible, at least partially, for the increased sialidase activity on the outer membrane of the platelets from the PT patients, and it induced desialylation of the platelet surface. Measurement of total sialidase activity after permeabilization indicated that NEU1 was not only over-expressed but was also released on the platelet surface.

We detected little sialidase activity in the blood serum from either the patients or the control group (Additional file 1: Figure S2).

### GPIbα desialylation is associated with increasing apoptosis of platelets

**Fig. 4:**
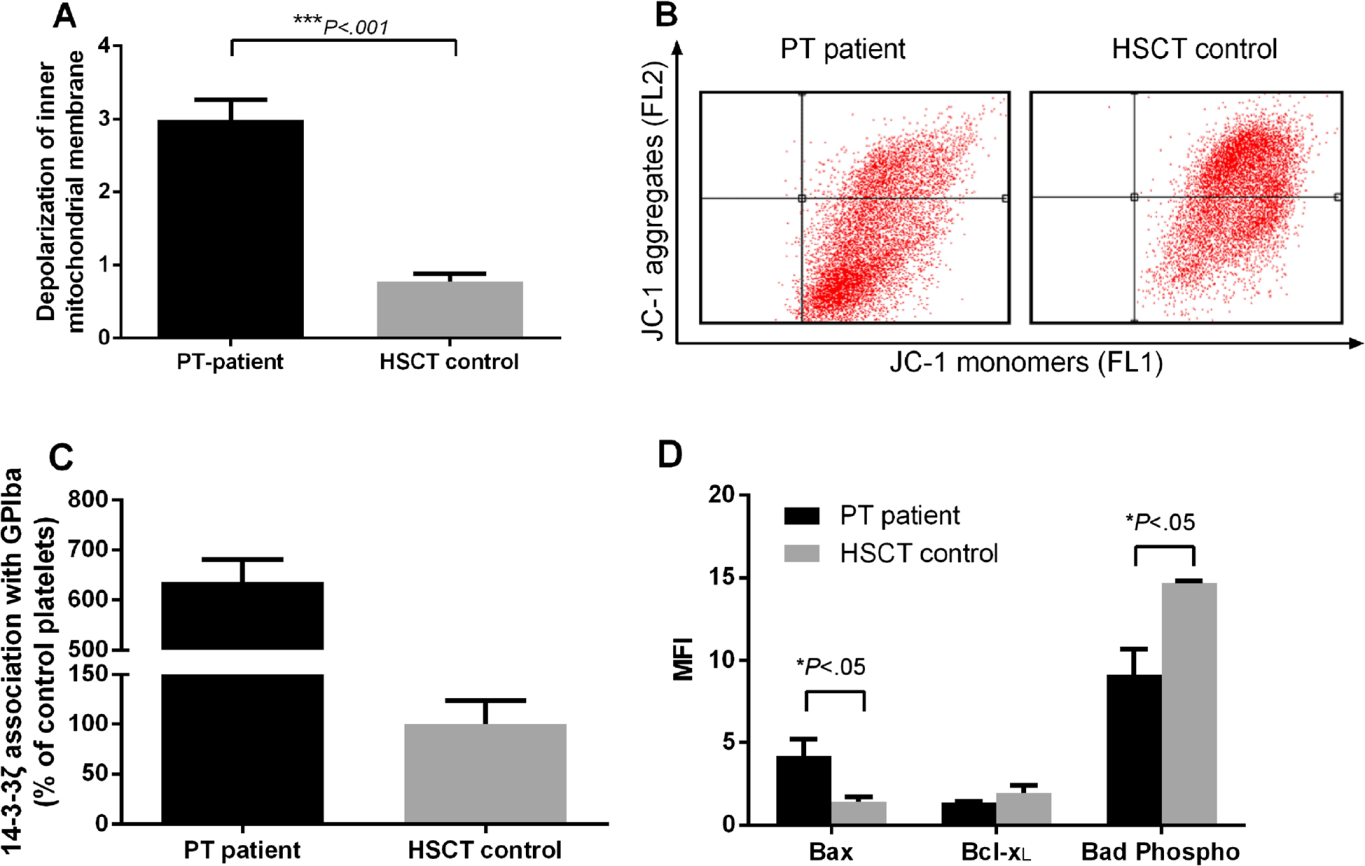
Platelets from the PT patients showed higher apoptosis. **a** Histogram of ΔΨm. The data are expressed as the ratio of fluorescent platelets in the lower-right quartile to those in the upper-right quartile. **b** ΔΨm as a measurement of JC-1 binding using flow cytometry. **c** 14-3-3ζ, associated with GPIbα, measured by immunoprecipitation. **d** Activated Bad, Bax, and Bcl-xL were analyzed by flow cytometry in platelets from the PT patients and the control group

To investigate the clearance mechanism of the platelets in the peripheral blood, we analyzed the apoptosis of the platelets. The platelets were incubated with JC-1 dye to detect the depolarization of the inner mitochondrial membrane because it is a signal of intrinsic apoptosis pathway initiation. Platelets from the PT patients showed a remarkably larger ΔΨm than in the control group (2.992 ± 0.2753 vs. 0.7764 ± 0.1121, *P* = .028) (Fig. 4a), indicating an increase in intrinsic apoptosis (Fig. 4a). A correlation between the increased levels of RCA-I binding and elevated ΔΨm was also observed (Spearman’s correlation *ρ* = 0.585, *P* = .017).

To investigate whether the increase in apoptosis initiation was associated with GPIbα desialylation, we measured GPIbα association with 14-3-3ζ proteins. The platelets from the PT patients exhibited an approximately sixfold higher association rate between GPIbα and 14-3-3ζ, compared to the platelets from the control group (635 ± 45.23 % of the control, *P* < .001) (Fig. 4c). Moreover, analysis of Bad confirmed that loss of this association increased the dephosphorylation of Bad. To confirm that Bad dephosphorylation played a role in this procedure, two Bcl-2 family members were investigated. Flow cytometric analysis with antibodies against the active epitope of Bax and the active conformation of Bcl-xL revealed that Bax and Bcl-xL changed in parallel with the phosphorylation of Bad (Fig. 4d). Platelets from the PT patients showed a twofold increase in active Bax (*P* = .027) and a tendency toward reduction in active Bcl-xL (*P* = .059).

### Macrophages prefer to phagocytize desialylated platelets from patients with PT in vitro

**Fig. 5:**
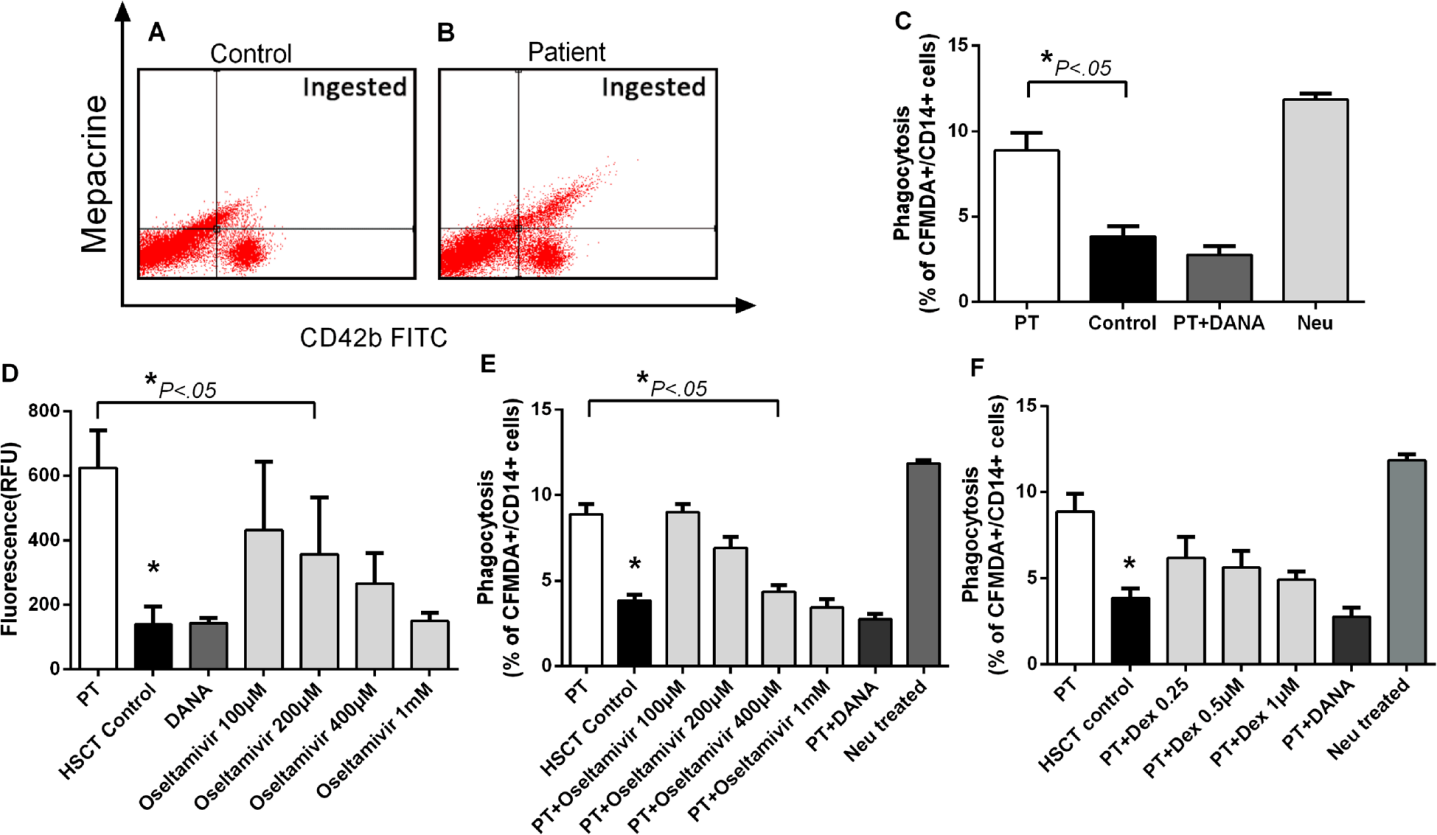
In vitro phagocytosis of platelets and the influence of oseltamivir. Stimulated THP-1 cells were incubated with platelets. **a** Control response. **b** Patients’ response. **c** Phagocytosis rates of the PT patients, HSCT controls, and PT patients with DANA. Platelets from the healthy controls were treated with Neu to provide a positive control. **d** Effects of different concentrations of oseltamivir on sialidase activity on the platelet surface. **e** Effects of different concentrations of oseltamivir on the phagocytosis of platelets. **f** Effects of different concentrations of dexamethasone on the phagocytosis of platelets. The concentration of DANA is 1 mmol/L

Next, we analyzed the phagocytosis of the platelets in vitro to investigate further the clearance mechanism. Macrophages were differentiated from THP-1 cells by stimulating them with PMA. The platelets from the PT patients exhibited a twofold increase in the phagocytosis ratio, compared to platelets from the control group (*P* = .045; Fig. 5c). Consistent with the platelets from the patients, desialylated normal platelets that were incubated with 10 mU of *Neu* showed an even higher phagocytosis ratio (12.4 ± 0.55 %, Fig. 5c). This process could be inhibited by 2,3-didehydro-2-deoxy-N-acetylneuraminic acid (DANA) (*P* < .05).

### Risk factors for PT

In the univariate logistic regression, a history of GvHD, CMV reactivation after allo-HSCT, and decreased SNA binding were associated with PT (*P* < .10). In the multivariate logistic regression analysis, the MFI of SNA binding (odds ratio [OR], 8.137; 95 % confidence interval [CI], 2.364 to 28.005; *P* = .001) and a history of GvHD (OR, 4.056; 95 % CI, 1.171 to 14.050; *P* = .027) emerged as the primary independent risk factors for PT (Table 2). There was no association found between a history of GvHD and SNA binding in this study.

**Table 2:** Univariate and multivariate analysis of risk factors of PT

Risk factors	Univariate analysis of *P* value*	Multivariate analysis		
		OR	95 % Cl	*P* value**
Demographics				
Gender, female vs. male	.580			
Age, per decade	.103			
Disease characteristics				
Diagnosis, acute leukemia vs. other	.070			
Transplantation characteristics				
Donor type, PMRD vs. others	.246			
Status, high risk vs. standard risk	.967			
Transplantation-related complications				
History of severe GvHD	.017	4.056	1.171–14.050	.027
CMV infection after HSCT	.016			
Desialylation levels				
MFI of SNA^a^	<.001	8.137	2.364–28.005	.001

*Abbreviations*: *OR* odds ratio, *CI* confidence interval, *HSCT* hematopoietic stem cell transplantation, *PT* prolonged isolated thrombocytopenia, *PMRD* partially matched related donor, *GvHD* graft-versus-host disease, *CMV* cytomegalovirus

*P < .10 in univariate analysis; **P < .05 in multivariate analysis

^a^The MFI of SNA was separated into 2 groups according to the receiver operating characteristic (ROC) cutoff point (23.5)

### Oseltamivir decreased the phagocytosis of desialylated platelets by macrophages

Because we identified the involvement of the sialidase NEU1 in the pathogenesis of PT, we hypothesized that the sialidase inhibitor oseltamivir might inhibit NEU1 activity and reduce the clearance of platelets. To confirm our hypothesis, oseltamivir was added to the sialidase activity assay and phagocytosis assay. In the sialidase activity assay, oseltamivir at different concentrations (100 μM, 200 μM, 400 μM and 1 mM) induced a decrease in sialidase activity on the PT platelet surfaces in a dose-dependent manner (Fig. 5d). The difference was significant (*P* < .05) when the concentration of oseltamivir was greater than 200 μM, and the inhibition rate was 33 % at 400 μM. These data were consistent with the results using DANA. In the phagocytosis assay, oseltamivir treatment reduced the rate of platelet phagocytosis in a dose-dependent manner after 30 min of incubation (Fig. 5e). The difference was significant (*P* < .05) when the concentration of oseltamivir was greater than 400 μM, protecting approximately 20 % platelets from phagocytosis.

Because PT shares some similarities with immune thrombocytopenia, corticosteroids such as dexamethasone have been administered to treat PT after transplantation, according to therapy for ITP. Therefore, we used dexamethasone for treatment comparison. The results showed a dose-dependent reduction in the phagocytosis ratio of platelets with different concentrations of dexamethasone (Fig. 5f). However, this difference was not statistically significant.

## Discussion

Our results indicated for the first time that desialylation contributed to the pathogenesis of PT after transplantation and suggested two possible mechanisms (apoptosis and phagocytosis) by which desialylation contributes to the pathogenesis of PT. We also observed that oseltamivir could inhibit the catalytic activity of sialidase on the platelet surface and could reduce the phagocytosis of platelets by macrophages.

PT has been reported to be associated with an increased risk of bleeding [52], and it has been related to life-threatening complications and poor overall survival [2]. Several risk factors, including the source of stem cells, the CD34+ cell count, disease status, GvHD, CMV infection, and the bone marrow vascular microenvironment, have been proposed to be associated with PT after allo-HSCT [1, 2, 5, 9, 53]. By multivariate analysis, we found that a history of GvHD was an independent risk factor for PT after allo-HSCT. However, after the exclusion of patients with causes described above, there were still some PT patients showing no direct cause of thrombocytopenia. We previously found that PT after allo-HSCT might be related to defects in megakaryocyte maturation, probably due to an impaired bone marrow immune and vascular microenvironment [1, 9, 11]. However, the disruption of the bone marrow environment cannot explain the clinical manifestations by which some PT patients showed similar presentations to ITP patients, such as abnormalities in T cells mediated immunity [54]. Moreover, there was evidence demonstrating that increased platelet turnover played a role in the pathogenesis of PT [2]. Elevated platelet turnover could be found in these patients, including increased MPV and GCI [2]. Allo- or autoantibodies against platelets can obviously accelerate the destruction of platelets, which is the main pathogenesis of ITP [55]. However, recipients after allo-HSCT have different characteristics from non-transplant patients with thrombocytopenia, including ITP, because immune reconstitution has not been accomplished [56], and most of the recipients received strong immune suppression therapy (CsA, MMF, glucocorticoids, or anti-CD20/CD25 monoclonal antibodies). Due to allo-HSCT itself and immunosuppression therapy after transplantation, the main lymphocyte populations (including CD3+ T cells, CD4+ T cells, CD8+ T cells, and B cells) required 4 to 6 years post-transplantation to return to age-matched control levels, with absolute numbers of B cells increasing gradually and reaching normal levels more than 1 year after HSCT [56, 57]. Therefore, the production of antibodies was strongly inhibited when samples were collected in this study [57]. Some anti-CMV antibodies can increase the platelet turnover, as previously described [58, 59], but the positive rate of CMV antibody was obviously lower than that of DNA quantified by PCR and of pp65 antigen detected by FCM [60]. Thus, it needs to be elucidated whether anti-platelet antibodies participate in the occurrence of PT after allo-HSCT, like they do in the pathogenesis of ITP. A previous study found no association between anti-platelet antibody response and PT after allo-HSCT [2]. Therefore, we hypothesized that some antibody-independent platelet clearance participated in the pathogenesis of PT, such as the loss of sialic acids found in cooled platelets.

Because sialic acids can act as “self-associated molecular patterns” (SAMPs) and inhibit immune activation [27], desialylation can induce the activation of innate immune cells and lymphocytes, as well as trigger the phagocytosis of proteins and cells [20, 27, 45, 61]. Desialylation is known to lead to the sequestration of platelets from the blood by the reticuloendothelial system, and it has been correlated with a reduction in the platelet life span [62, 63]. It has been demonstrated to be a key step in the clearance of transfused refrigerated platelets [21, 28], and it also plays a role in the pathogenesis of thrombocytopenia during sepsis and in some parasitic infections [62, 64]. Consistent with these findings, our data showed that desialylation also participated in the pathogenesis of PT after transplantation.

The significantly higher concentration of sialic acid levels in the serum of PT patients might not only be attributed to platelet desialylation but might also result from inflammatory conditions or vascular damage, because both the patient and control groups showed higher levels than the healthy control group. Although platelets are one of the sources of sialic acids [65, 66], glycoproteins and other cellular components in the peripheral blood or from tissue can be desialylated, leading to elevated sialic acid concentration in plasma [27]. Serum sialic acids have been demonstrated to be a general indication of the “acute phase response” and related to vascular damage in patients with diabetes and cardiovascular disease [25, 67]. The higher sialic acids in the plasma of HSCT recipients than in the healthy controls could be attributed to HSCT therapy and complications, because most recipients of allo-HSCT experience conditioning regimens with high-dose chemotherapy, engraftment of donor cells, and possibly aGvHD, TMA, infections, and drugs [30, 34].

Tracing the enhanced desialylation to the platelet surface plasma membrane, we detected elevated sialidase activity on platelets from the PT patients, whereas the serum sialidase activity was undetectable in all of the groups in our study (Additional file 1: Figure S1), and platelets from the control group showed little sialidase activity on the surface. This finding suggested that desialylation was not mediated by serum neuraminidase from pathogens.

In human tissues, NEU1 generally exhibits the strongest expression of the human neuraminidases (10–20 times greater than those of NEU3 and NEU4) [68], and our data from the platelets were consistent with this. We detected surface over-expression of NEU1 in the PT patients with platelet desialylation, supporting the hypothesis that desialylation of platelets is caused by NEU1 over-expression on platelet surfaces and not by exogenous neuraminidases from pathogens.

Although NEU1 is typically located in the lysosomes, recent studies have revealed the subcellular localization of NEU1 sialidase to the plasma membrane as well as within lysosomes under conditions of cell stimulation [69], allowing NEU1 to desialylate glycoproteins on the cell surface or in the extracellular environment [70]. On the platelet surface, NEU1 expression is elevated after refrigeration, and it desialylates platelet surface glycoprotein, leading to platelet clearance from the peripheral blood [28].

GPIbα is a membrane protein highly glycosylated with plenty of sialic acid residues at the end of its glycan chains. Desialylation of GPIbα could induce clustering and might result in the redistribution of 14-3-3ζ in the cytoplasm and apoptosis initiation [48, 71].

Platelet apoptosis has been reported in a number of physical and pathological settings, including chemical stimuli, high shear stress, ITP pathogenesis, and *Helicobacter pylori* bacterial infection [72]. The intrinsic mitochondria-dependent pathway of apoptosis in the platelets has been well documented, and a number of key parameters of the intrinsic pathway of apoptosis have been induced in the platelets in response to different triggers, including ΔΨm depolarization; the expression, activation, and translocation of pro-apoptotic Bax, Bak, and Bid to the mitochondria; the release of cytochrome c into the cytosol; and the activation of caspase-9 [72].

GPIbα is associated with 14-3-3ζ proteins, which in addition to contributing to GPIbα signaling via phosphatidylinositol 3-kinase (PI3K) during vWF ligation, also control the dephosphorylation of Bad, an upstream element in the intrinsic apoptosis pathway. 14-3-3ζ sequesters cytosolic Bad, thereby protecting its phosphorylated, dormant state against activation [48, 73, 74]. Because the association of 14-3-3ζ with GPIbα might disturb its association with Bad and induce apoptosis, we first investigated apoptosis in the platelets from the PT patients, and we speculated that there were associations among GPIbα desialylation, clustering, 14-3-3 protein redistribution, and apoptosis. We clarified that the apoptotic markers along the intrinsic pathway were present in different profiles in the PT patients compared to the HSCT control group, indicating an increase in apoptosis. This change was related to the redistribution of 14-3-3ζ, indicating an association between desialylation and enhanced apoptosis.

Desialylation exposes β-galactose and β-GlcNAc at the end of the glycan chains, which could be recognized by their corresponding receptors on liver cells and macrophages, respectively [24, 29]. These receptors, also called lectins, are carbohydrate-binding proteins containing a carbohydrate recognition domain (CRD) that binds with high specificity to the outermost sugars of glycans, as well as glycosphingolipids [23]. Mac-1 (CR3, αMβ2; CD11b/CD18) is a β2 integrin expressed by macrophages, and previous studies have shown that desialylated and clustered GPIbα on chilled platelets was the counter-receptor for Mac-1, leading to their clearance by phagocytosis [24, 49].

Our data showed an increased phagocytosis ratio of platelets from the PT patients, whereas DANA could diminish phagocytosis, which indicated that desialylation occurred continuously in the circulation and sialidase inhibitor could help to reduce desialylation in vitro. However, it has also been reported that desialylation led to phagocytosis by liver cells via Ashwell receptors in chilled platelets [29] although whether a similar mechanism is involved in PT pathogenesis remains to be determined.

We surmised that if desialylation was associated with platelet apoptosis and phagocytosis, sialidase inhibitors might diminish platelet clearance and facilitate platelet survival. Thus, we treated the samples with oseltamivir and found that it could also inhibit human sialidase activity and reduce the phagocytosis of platelets in vitro. Our data demonstrated that oseltamivir could also inhibit human sialidase activity. Upon addition to the phagocytosis test system, oseltamivir also showed inhibition of phagocytosis. The effect of oseltamivir on the phagocytosis of platelets from the control group was unknown, but because few platelets from the control group were consumed by the phagocytes, this uncertainty did not affect our conclusion that oseltamivir could ameliorate the phagocytosis of platelets from PT patients. This observation indicated a novel strategy for the management of PT after allo-HSCT, because oseltamivir can be administered to PT patients who show no reaction to corticosteroids. Moreover, because NEU1 expression indicated an antibody-independent platelet clearance mechanism, which is a condition under which corticoids might not work well, it could be speculated that NEU1 over-expression in PT patients might be associated with resistance to corticoids, and these patients might benefit from sialidase inhibitors, such as oseltamivir. Because the specific target of oseltamivir was not verified in this study, further effort should be undertaken to investigate the sialidase that is inhibited by oseltamivir, because in this study, it was oseltamivir itself that exhibited the inhibition and not oseltamivir carboxylate, which is the main metabolized form. Previous studies have shown that oseltamivir could inhibit NEU1 and could impede human pancreatic cancer growth [75]. Hata et al. reported that oseltamivir carboxylate scarcely affected the activities of any of the sialidases [76].

Post-transplant thrombocytopenia is frequently caused by multiple factors, and it is frequently difficult to determine the exact cause of thrombocytopenia after allo-HSCT, such as infection, GvHD, drugs, impaired bone marrow microenvironment, and desialylation. The patients with desialylated platelets in this study showed no explicit cause of thrombocytopenia, and they exhibited elevated platelet turnover. Some patients might share similar characteristics to patients with ITP, such as T cell abnormalities [54, 77].

The present study demonstrated that desialylation on the platelet surface was significantly higher in patients with PT after allo-HSCT. Both NEU1 expression and activity on the platelet surface were significantly elevated in the PT patients. Furthermore, our data indicated that platelet desialylation played a role in PT pathogenesis because desialylation was correlated with increased platelet apoptosis and phagocytosis. It was shown that oseltamivir could inhibit the desialylation activity on the platelet surface and subsequently decrease the phagocytosis of platelets, indicating a novel candidate strategy for the management of PT after allo-HSCT.

## Additional file

Additional file 1:
**Supplemental figures of serum sialidase activity and confirmation of desialylated glycoprotein.** (PDF 96 kb)
