# Supplementary material for: Desialylation is associated with apoptosis and phagocytosis of platelets in patients with prolonged isolated thrombocytopenia after allo-HSCT
Source: J Hematol Oncol. 2015 Oct 23;8:116. doi: 10.1186/s13045-015-0216-3 (PMC4619537; doi:10.1186/s13045-015-0216-3)
Supplement: Additional file 1: — Supplemental figures of serum sialidase activity and confirmation of desialylated glycoprotein. (PDF 96 kb) [file 13045_2015_216_MOESM1_ESM.pdf]

Supplemental Figures

Supplemental figures of serum sialidase activity and confirmation of desialylated glycoprotein.

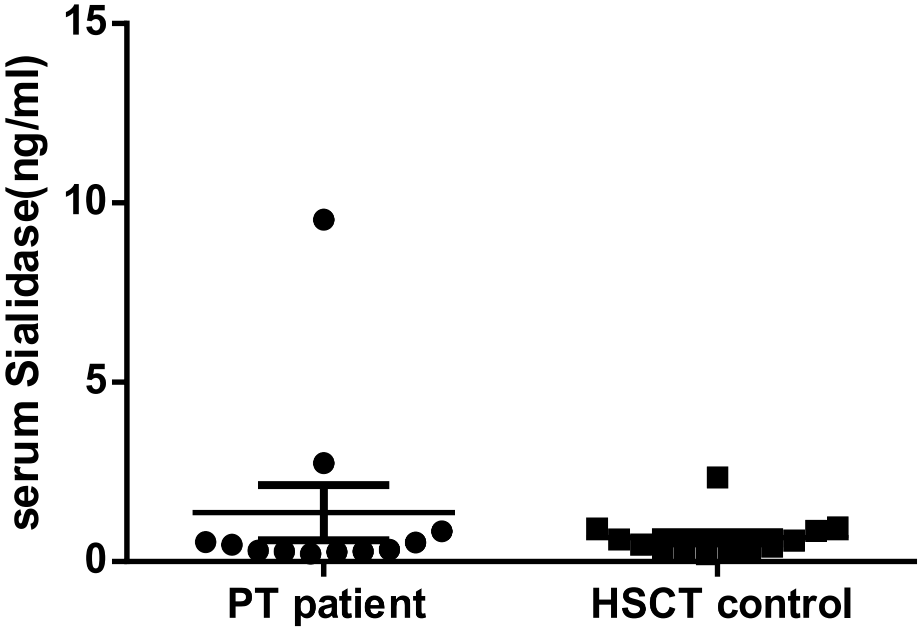

Figure S1. Serum sialidase measured by ELISA in PT patients and HSCT control group.

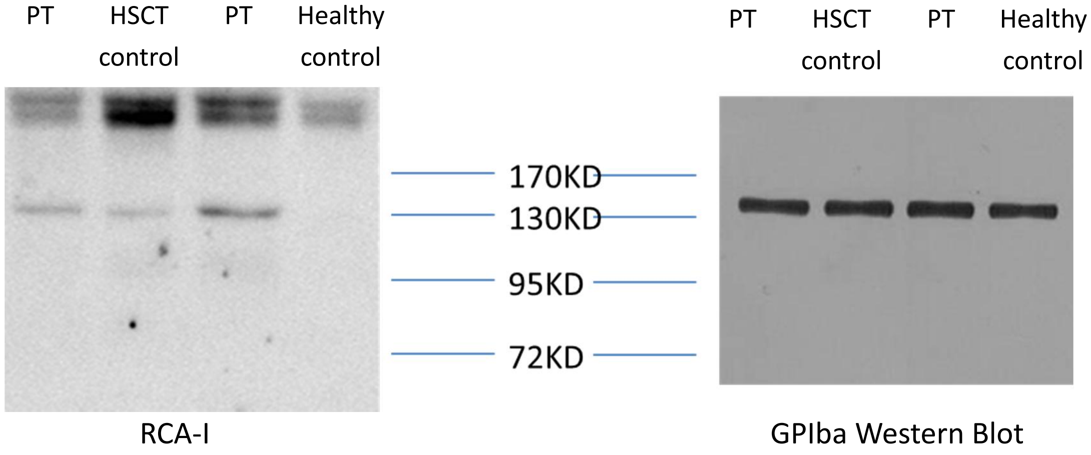

Figure S2. Desialylated glycoprotein Immunoblotting.
